# Neu-medullocytes, sialidase-positive B cells in the thymus, express autoimmune regulator (AIRE)

**DOI:** 10.1038/s41598-018-37225-y

**Published:** 2019-01-29

**Authors:** Shigeko Kijimoto-Ochiai, Keiko Kamimura, Toshiaki Koda

**Affiliations:** 1 Faculty of Advanced Life Science, Hokkaido University, N21 W11, Kitaku, Sapporo 001-0021 Japan; 2 Present Address: Life Space COSMOS, Hirosaki, 036-8222 Japan

## Abstract

Neu-medullocytes, which were previously identified and named by our group, are sialidase (neuraminidase)-positive B cells that express immunoglobulin and Mac-1 in the mouse thymus. Recently, B cells that migrated into the thymus were reported to express autoimmune regulator (AIRE) and to contribute to self-tolerance. We sought to determine whether Neu-medullocytes also express AIRE. We obtained positive results by triple staining Neu-medullocytes for *in situ* sialidase activity, anti-AIRE, and either anti-IgG or anti-IgM antibodies and observing the staining with confocal microscopy. Additional molecules including CD5, IgM, major histocompatibility complex (MHC) Class II, and neuraminidase 1 (NEU1) were found in sialidase-positive cells independently. The real-time PCR results suggest that the primary sialidase in AIRE-positive cells is neuraminidase 2 (NEU2). Furthermore, some of the AIRE-positive medullary thymic epithelial cells also clearly showed sialidase activity when a triple staining of sialidase activity, anti-AIRE, and *Ulex europaeus* agglutinin-1 (UEA-1) was performed. Neu-medullocytes may present *Aire*-dependent antigens for negative selection. We discuss the negative selection steps in consideration of sialidases and sialic acids.

## Introduction

Neu-medullocytes are sialidase- (neuraminidase-) positive B cells that express immunoglobulin and Mac-1 in the mouse thymus^1,2^. The cells were identified in 2004^1^ and were so-named in 2008^2^ because they were newly discovered neuraminidase (NEU)-positive cells in the mouse thymus and because they exist primarily in the corticomedullary junction or in the medulla^2^. Neu-medullocytes can be stained with blue fluorescein by using 5-bromo-4-chloro-3-indolyl-5-α-D-N-acetylneuraminic acid cyclohexylamine salt (X-NANA), an artificial sialidase substrate. When the bond between X (5-bromo-4-chloro-3-indolyl 5-acetamido-3,5-dideoxy-OH) (or very similar compound^3^) and NANA (N-acetylneuraminic acid) is hydrolyzed by sialidase, X becomes an insoluble material with blue fluorescence^1,3^. Thus, X-NANA is used as an artificial substrate for identifying the *in situ* activity of sialidases, especially that of NEU2.

Sialidases (EC 3.2.1.18) are a family of exo-glycosidases that remove terminal sialic acid residues from the glycans of glycoproteins, glycolipids, and oligosaccharides. These enzymes are widely distributed and are found in viruses, protozoa, bacteria, fungi, and vertebrates^4^. Four types of vertebrate sialidases, lysosomal NEU1, cytosolic NEU2, plasma membrane NEU3, and mitochondrial/lysosomal/intracellular membrane NEU4, are well established, and comprehensive reviews discussing them have been published^4,5^. However, recent studies in the last ten years have shown that “lysosomal” NEU1 exists in the plasma membrane in many cases under some physiological conditions, and it has emerged as a key actor involved in cell signaling regulation^5–7^. Recently, we have shown that NEU1 exists on the cell surface of mouse thymocytes whose natural substrate is CD5^8^.

Thymic B cells have been identified in humans^9^ and in mice^10^. In mice, 75% of thymic B cells were shown to be CD5^+^ and were not stimulated via surface Ig and IL-4 but required direct interaction with T blasts^11^. The circulation of B cells through the thymus from the periphery has also been reported, although the number of cells was small^12^. Recent studies demonstrated that B cells in the murine thymus can become activated, and it was shown that the autoreactive thymic B cells are efficient antigen-presenting cells (APCs) for cognate self-antigens during T cell negative selection^13^; B cells that migrate into the thymus express AIRE, upregulate MHC class II and CD80 expression, and act as APCs for negative selection^14^. B cell differentiation and the expression of AIRE were confirmed in the human thymus^15^; researchers analyzed the expression of AIRE and some tissue-restricted antigen (TRA)-genes and found support for the hypothesis that B cells are involved in negative selection^15^. *Aire* was found to be deficient in patients with an autoimmune disease^16,17^. It has become clear that, at least in part, *Aire* regulates the ectopic expression of TRAs in medullary thymic epithelial cells (mTECs)^18,19^. *Aire* expression is inherent to all mTECs but may occur at particular stage(s) and/or cellular states during their differentiation^20^. The expression of *Aire* in B cells in the thymus must play an important role. Thus, we asked whether Neu-medullocytes also express AIRE because Neu-medullocytes express immunoglobulin and Mac-1^1^, although it is not known whether these cells originate from circulating B cells^14^ or from progenitors within the thymus^13^. We stained mouse thymus cells with X-NANA, anti-AIRE, and anti-IgG or IgM and observed them using confocal microscopy. We then sought to determine whether AIRE^+^ mTECs also show sialidase activity. In the Discussion section, we consider the physiological functions of Neu-medullocytes and sialidase in the thymus.

## Results

### Antigens expressed in Neu-medullocytes as B cells: IgG, CD5, IgM, and MHC class II

**Figure 1:**
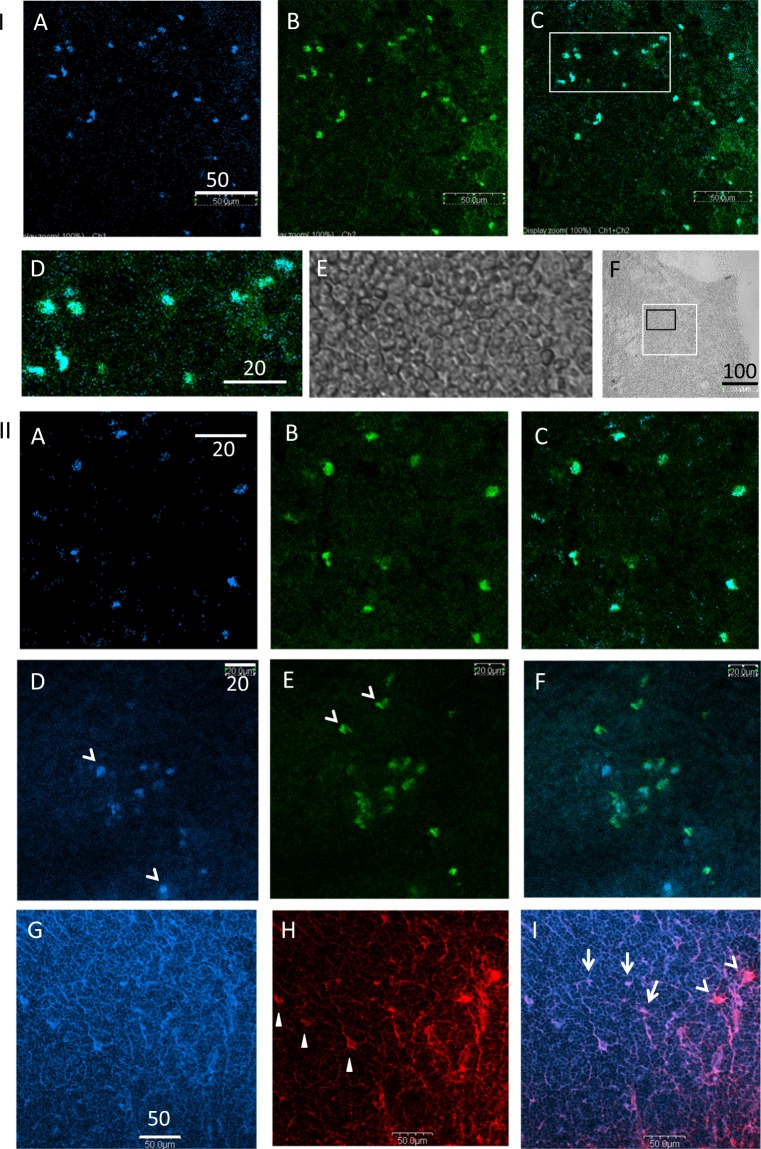
Antigens expressed in Neu-medullocytes as B cells: IgG, CD5, IgM, and MHC class II. (**I**) Reconfirmation of the expression of IgG on X-NANA-stained cells using F(ab′)_2_ fragment of anti-mouse IgG as detected by confocal microscopy. The thymus from a C57BL/6 mouse (male, 6 W) was used. (**A**) X-NANA-stained; (**B**) FITC-anti-mouse IgG; (**C** and **D**) merged (**A** and **B**). (**D** and **E**) enlarged area enclosed by a white square in (**C**) and its DIC (differential interference contrast) image, respectively. (**F**) DIC image including the areas from (**A**–**C**) (white square) and (**D**,**E**) (black square). Scale bars in (**A**,**D** and **F**) indicate 50 (for **A**–**C**), 20 (for **D** and **E**) and 100 µm (for **F**), respectively. (**II**) Other antigens expressed on X-NANA stained cells. The thymus sections from C57BL/6 mice were used for (**A**–**C**) (male, 6 W). The thymus sections from AKR mice (haplotype k) were used for (**D**–**F**) (male, 8 W) and for (**G**–**I**) (female, 5 W). (**A**,**D** and **G**) X-NANA stained; (**B**) FITC-anti-CD5 stained; (**C**) merged (**A** and **B)**; (**E**) FITC-anti-MHC class II (anti I-A^k^) stained; (**F**) merged (**D** and **E**); (**H**) R.R.-anti-IgM stained; (**I**) merged (**G** and **H**). The scale bars (μm) indicate 20 in A (for **A**–**C**), 20 in D (for **D**–**F**), and 50 in G (for **G**–**I**).

First, we reconfirmed that Neu-medullocytes are B cells^1^ and excluded the possibility of the binding of antibodies through Fc receptors. FITC-labeled F(ab′)_2_ fragment of anti-mouse IgG was used to staining cryostat sections of mouse thymus that were also stained with X-NANA (Fig. 1I). X-NANA-positive Neu-medullocytes (Fig. 1I,A) and FITC-anti-mouse IgG-stained cells (Fig. 1I,B) completely overlapped (Fig. 1I,C). The enlarged image (Fig. 1I,D) and its DIC image (Fig. 1I,E) are shown with at a lower magnification (Fig. 1I,F). Neu-medullocytes were reconfirmed to contain IgG and to be a kind of B cells. However, IgG positive cells do not always have X-NANA sialidase activity as shown in Supplementary Fig. S1.

Next, the expression of further CD5, MHC class II, and IgM antigens in X-NANA-sialidase positive cells was studied (Fig. 1II). CD5, a marker of the B1a-cell lineage in mice, was characterized as a thymic B cell marker because 75% of thymic B cells were CD5 and Mac-1 positive, whereas splenic B cells lacked CD5^10,11^. Figure 1II,A–C shows that almost all of the X-NANA positive cells (Fig. 1II,A) were also CD5 positive (Fig. 1II,B and C). Thus, Neu-medullocytes were again confirmed to be B cells in the thymus. Next, the expression of MHC class II was studied because high levels of MHC class II have been reported in migrated B cells^14^, in thymic B cells that developed from progenitors within the thymus^13^, and in mTECs^21^. Figure 1II,D–F show MHC class II- positive (Fig. 1II,E, green) and X-NANA-positive (Fig. 1II,D, blue) cells, although some cells were MHC class II-positive but X-NANA-negative or only faintly X-NANA-positive (Fig. 1II,E, indicated by arrowheads) and vice versa (Fig. 1II,D, indicated by arrowheads). IgM, expressed in circulating mouse B cells^14^ and also in the medulla of human thymus^15^, was stained with Rhodamine Red^TM^-X (R.R.)-(Fab′)_2_-anti-IgM (Fig. 1II,H). Some cells were IgM/X-NANA-positive (indicated by arrows in Fig. 1II,I) and some larger cells were also positive for both (indicated by triangles in Fig. 1II,H), but the largest cells were X-NANA-negative (indicated by arrowheads in Fig. 1II,I). These findings indicate that Neu-medullocytes are B cells that contain IgG and CD5 and that some are also positive for MHC class II antigens and IgM.

### Detection of AIRE in Neu-medullocytes by confocal microscopy

**Figure 2:**
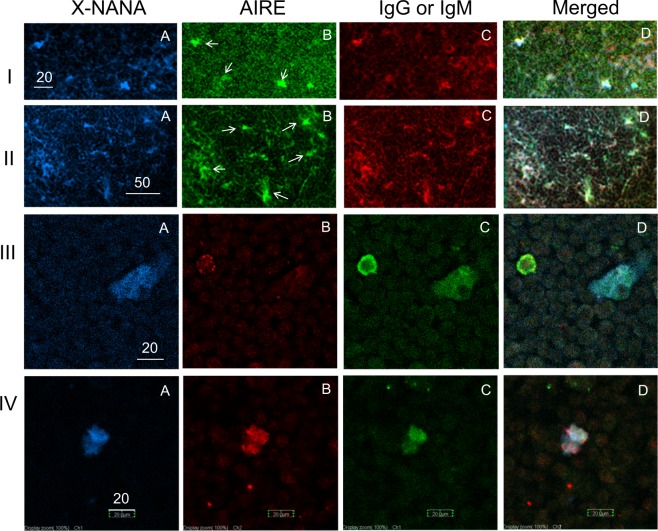
Expression of AIRE in Neu-medullocytes. (**I** and **II**); thymus sections from AKR mice (male, 12 W) were used. (**A**) Stained with X-NANA; (**B**) stained with anti-AIRE (anti-AIRE and FITC-conjugated goat anti-rabbit IgG); (**C**) stained with R.R.-F(ab′)_2_-anti-mouse IgG (I) or R.R.-F(ab′)_2_-anti-mouse IgM (II); (**D**) merged (**A**–**C**). The AIRE-positive cells that are indicated by arrows in B are X-NANA and IgG or IgM triple-positive cells (white) in D. (**III** and **IV**); These cells were prepared from three thymuses from C57BL/6 mice (male, 6 W) by enzyme digestion and separated into the total thymocyte (T fraction) (**III**) and E (E1 + E2) fractions (**IV**). (**A**) stained with X-NANA; (**B**) stained with anti-AIRE coupled with rhodamine (TRITC)-conjugated donkey anti-rabbit IgG); (**C**) stained with FITC-F(ab)_2_-donkey anti-mouse IgG; (**D**) merged (**A**–**C**). Scale bars are 20, 50, 20 and 20 μm for (**I**, **II**, **III** and **IV**), respectively.

Next, we asked whether Neu-medullocytes express AIRE. A thymus section was triple stained with X-NANA, FITC-anti-AIRE and R.R.-anti-IgG or -anti-IgM antibodies (Fig. 2I,II). Most of the AIRE-positive cells from AKR mice (Fig. 2I,II, indicated by arrows in B) were X-NANA-sialidase positive (Fig. 2I,A,II,A) and were IgG (Fig. 2I,C) or IgM (Fig. 2II,C) positive. The white cells in the merged image of all three colors are X-NANA-, AIRE-, and IgG- or IgM-positive cells (triple positive). Figure 2III,IV show the results of assays using fractionated cells that were obtained by enzyme treatments of the thymus^22^, as described in the Methods section. We found triple positive cells in the E fraction (IV); however, triple positive cells were rarely found in the T fraction (III), although we found X-NANA and IgG positive cells in the T fraction (Supplementary Fig. S2). This result is consistent with the low expression level of *Aire* in the T fraction observed in RT-PCR analysis (as shown in a later section). The size of IgG-positive cells in the T fraction was larger than that of T cells, and the size of AIRE-positive cells in the E fraction appears to be the same or slightly larger than that of IgG-positive cells (see Supplementary Fig. S2).

AIRE^+^ mTECs are known to act as APCs that express the ectopic tissue-specific antigens induced by *Aire*, but they do not contain IgG or IgM. We showed, in Fig. 2, that some of the AIRE-positive cells are clearly IgG- or IgM- and X-NANA-positive (i.e. these cells are Neu-medullocytes as B cells). Thus, it became clear that at least some Neu-medullocytes express AIRE.

### Some mTECs showed X-NANA sialidase activity

**Figure 3:**
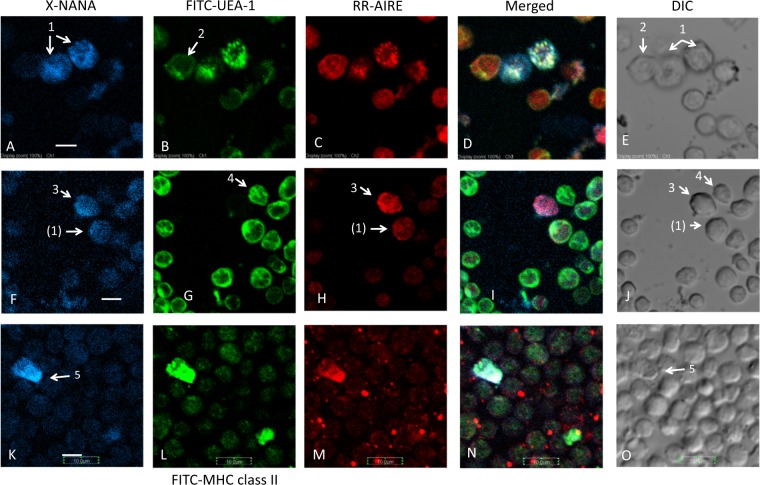
X-NANA, UEA-1 or MHC class II, and AIRE staining of isolated thymic stromal cells. These cells were prepared from three thymuses (C57BL/6 male, 6 W) by enzyme digestion, and the epithelial rich fraction (E2) was used. Image groups (**A**–**E**) and (**F**–**J**) are stained with X-NANA, FITC-UEA-1, and anti-AIRE coupled with Rhodamine Red^TM^-X secondary antibody but shown different fields. Image group (**K**–**O**) was stained with FITC-anti-MHC class II instead of FITC-UEA-1. (**A**,**F** and **K**), X-NANA (blue); (**B**,**G** and **L**), UEA-1 or MHC class II (green); (**C**,**H** and **M**), AIRE (red). (**D**,**I** and **N**) are the merged images of the three colors; (**E**,**J** and **O**) are DIC images. Scale bars in A, F and K are 5 μm. Cell 1 was X-NANA, UEA-1 and AIRE positive cell, cell 2 was X-NANA negative, UEA-1 and AIRE positive cell, cell 3 was X-NANA and AIRE positive, but UEA-1 negative cell, cell 4 was X-NANA and AIRE negative but UEA-1 positive, and cell 5 was X-NANA, MHC class II and AIRE positive. Cell (1) expressed weaker X-NANA sialidase activity than that of cell 1.

Next, we asked whether mTECs exhibited sialidase activity. A thymic stromal cell fraction (E2 fraction) was stained with X-NANA, FITC-UEA-1, and anti-AIRE coupled with Rhodamine (TRITC)-labeled secondary antibody (Fig. 3). UEA-1 preferentially binds to the terminally fucosylated type II blood group H sequence [Fucα1-2 Galβ1-4GlcNAc] with a cross-reactivity toward the Lewisy sequence [Fucα1-2 Galβ1-4(Fucα1-3)GlcNAc]^23^. The UEA-1-reactive ligand was expressed among mouse thymic epithelial cells and a subset of the mature medullary thymocytes^24^. We found that at least four types of cells were differentially stained with these reagents. In Fig. 3A–E, the cell indicated by arrow 1 was X-NANA (blue)-, UEA-1 (green)-, and AIRE (red)-positive (suggesting that this cell is a sialidase positive mTEC), and cell 2 was an X-NANA-negative and UEA-1- and AIRE-positive cell (suggesting that this cell is a sialidase negative mTEC). In Fig. 3F–J, cells 3 and 4 exhibited opposite characteristics: cell 3 was X-NANA- and AIRE-positive but UEA-1-negative (suggesting that this cell is not an mTEC but is a B cell lineage cell or that this cell is an immature or aged mTEC), whereas cell 4 was UEA-1-positive but AIRE- and X-NANA-negative (suggesting that this cell is an AIRE-negative mTEC). Cell 3 was stained evenly by anti-AIRE, whereas most other cells (e.g., cell (1) in Fig. 3H, or cell 1 in Fig. 3C) were stained by anti-AIRE only at the nucleus with several particles. This staining pattern is similar to those from a previous report^20^. The X-NANA stain of cell (1) in Fig. 3F was weaker than that of cell 1 in Fig. 3A. Thus, the three-color-merged image (Fig. 3I) did not show white. Cell 5 in Fig. 3K–O was stained by X-NANA, FITC-MHC class II and the R.R.-AIRE system. Though it is not apparent whether this triple positive cell is an mTEC or a Neu-medullocyte, the shape of cell 5 resembles those of established mTEC cell lines^25^. The results showed that at least some AIRE- and UEA-1-positive mTECs (cell 1) expressed X-NANA sialidase activity.

### mRNA expression of *Aire* and four sialidases in the fractionated thymus cells

**Figure 4:**
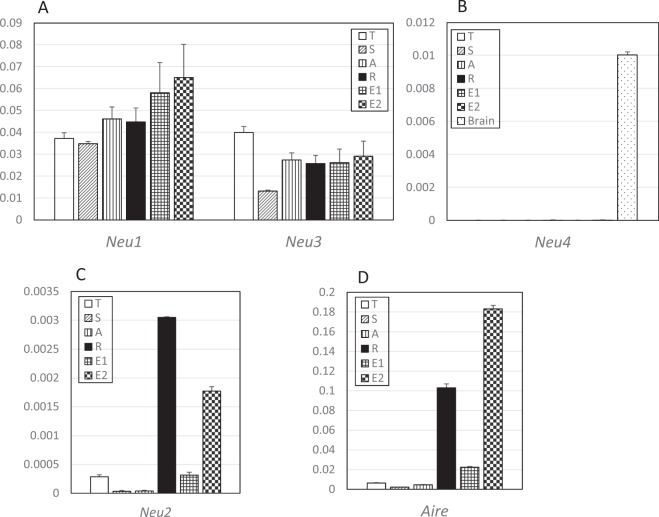
Relative expression levels of *Aire* and the four sialidases in fractionated cells from C57BL/6. (**A**) relative expression levels of *Neu1* and *Neu3* in total thymocytes (T, white bar), T-single cell (S, diagonal lined bar), T-aggregated cell (A, vertical lined bar), residual non-T (R, black bar), first enzyme treated (E1, grid bar), and second and third enzyme treated fractions (E2, checked pattern bar) from C57BL/6 mice (male, 6 W) compared to the expression levels of β-actin. (**B**–**D**) relative expression levels of *Neu4*, *Neu2*, and *Aire* in the six cell fractions. Total brain RNA was used as the positive control in the reaction of *Neu4* (dotted bar). All values are the averages of duplicates.

Next, to investigate the relationship between *Aire* and sialidase expression, the mRNA expression of *Aire* and four sialidases was studied with real-time PCR, using fractionated thymus cells. The expression pattern of *Aire* mRNA resembled that of *Neu2* sialidase (Fig. 4): *Aire* and *Neu2* were expressed mainly in the residual (R) and E2 (epithelial rich) fractions. Although R was further fractionated into E1 and E2, *Neu2* expression in R was higher than in either the E1 or E2 fractions. On the other hand, *Aire* expression in E2 was higher than that in R. This result is likely due to the enriched mTECs in the E2 fraction. Although *Neu1* and *Neu3* were also expressed in the R and E2 fractions, they were also expressed in the total T, single T, and aggregated T fractions. *Neu1* was also expressed at higher levels in the E2 fraction than in the R fraction like in the case of *Aire*. Thus, among the four types of sialidases, *Neu1* and *Neu2* appear to have distribution patterns similar to that of *Aire*. This result suggests that the sialidases expressed in the AIRE^+^ cells are likely to be primarily NEU2, with some NEU1.

### NEU1 sialidase was detected in Neu-medullocytes

**Figure 5:**
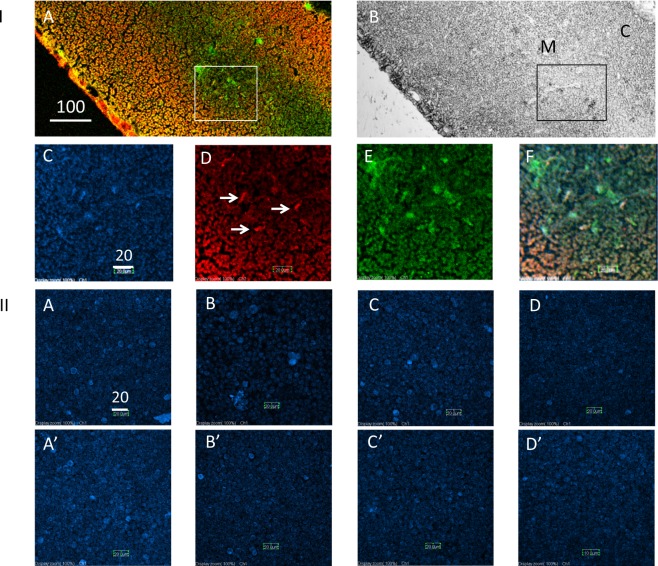
Expression of NEU1 sialidase on Neu-medullocytes and the effects of sialidase inhibitors. (**I**) Detection of NEU1 on Neu-medullocytes by anti-NEU1 antibody. The triple stained thymus section from AKR mouse (male, 5 W) was observed at a low magnification by confocal microscopy using the merged image of X-NANA (blue), anti-NEU1 (red) and anti-mouse IgG (green) (**A**) or a DIC image (**B**). The area enclosed by the square in A was enlarged and is shown in C–F. (**C**) X-NANA (blue); (**D**) anti-NEU1 (red); (**E**) anti-mouse IgG (green) (**F**) merged C–E. Scale bar is 100 μm in A and 20 μm in C for C–F. NEU1-positive Neu-medullocytes are indicated by arrows in D. (**II**) Effect of sialidase inhibitors on X-NANA staining. Thymus total cells from C57BL/6 mice (female, 6 W) were prepared by enzyme treatment, as described in the Methods section, and treated with X-NANA in the presence of sialidase inhibitors (30 µM). Images with the same letter indicate duplicate results. A,A’: no inhibitor; B,B’: DANA; C,C’: NEU1-selective inhibitor C9-BA-DANA; D,D’: siastatin B. Scale bar in A is 20 µm for all images.

Next, we investigated the possibility of NEU1 as a candidate sialidase acting in Neu-medullocytes, because the *Neu1* gene is located in the MHC region^26^, and NEU1 moves to the plasma membrane from the lysosome according to physiological signals^27^. The thymus section in Fig. 5I was triple stained with X-NANA (Fig. 5I,C), anti-NEU1 (Fig. 5I,D) and anti-IgG (Fig. 5I,E). NEU1-positive Neu-medullocytes are indicated by arrows in Fig. 5I,D. These cells were distributed mainly in the medulla region as shown in Fig. 5I,A and B. However, as mentioned in the above section, the main sialidase in Neu-medullocytes is likely the cytosolic NEU2. Therefore, the effects of sialidase inhibitors on X-NANA staining were studied, using the general sialidase inhibitor 2-deoxy-2,3-dehydro-N-acetylneuraminic acid (DANA), the NEU1-selective inhibitor C9-butyl-amide-2-deoxy-2,3-dehydro-N-acetylneuraminic acid (C9-BA-DANA), and the NEU2 inhibitor siastatin B, with thymus cells obtained by enzyme treatments (Fig. 5II). Although we could not confirm the effect quantitatively, siastatin B (Fig. 5II,D,D’) seemed to inhibit X-NANA staining (compare Fig. 5II,A,A’ and D,D’) more than the other inhibitors because X-NANA stained blue cells were hardly observed in those images.

## Discussion

In this study, we addressed the question of whether Neu-medullocytes express AIRE and found that some of them do. In reverse, we found that some AIRE^+^ mTECs exhibited X-NANA sialidase-activity. These findings expand our understanding of “sialidase-positive cells” in the thymus.

Sialidase related reports on the thymus are described below. Cell-to-cell interactions are important in the function of the immune system, but sialic acids on the cell surface prevent interactions because of their negative charge. We assumed the existence of a natural sialidase on the cell surface that was membrane-bound and active at physiological conditions (at neutral pH), on the basis of the observation that sialidase-treated B cells formed large cell aggregations^28^. We searched for such a membrane-bound sialidase and found that the mouse thymus exhibited a high level of sialidase activity when compared with other organs, such as lymph nodes or the spleen^1^. The membrane-bound form of sialidase that shows a higher level of activity at a neutral pH than at an acidic pH is unique and is never found among the four vertebrate sialidases^4,5^. *Neu2* from the mouse thymus was cloned, sequenced, and shown to be a unique type of sialidase, *Neu2B*, that has 6 extra amino acids in the N-terminus^29^, and only this B type of *Neu2* was expressed in the mouse thymus^30^. COS cells transfected with cloned *Neu2B* showed high membrane-bound sialidase activity^30^. The biochemical characteristics of the sialidases in the thymus were studied^31^, and the mRNA expression and distribution patterns for the four types of sialidase in the thymus were studied^2,8^. “Neu-medullocyte”, a unique sialidase-positive B cell in the thymus, was found via histochemical examinations^1,2^. Neu-medullocytes were not found in SM/J mice, which are known to be a sialidase activity-deficient Neu1^a^ strain^32^ and show very low expression levels of *Neu*2^2^. In addition, the sialidase detected with X-NANA must be NEU2 because NEU2 has the highest ability to hydrolyze artificial substrates such as 4-methyl umbelliferyl 5-acetyl neuraminic acid (4MU-NANA) and X-NANA^31,33^. From these, we infer that the sialidases in Neu-medullocytes are NEU1 and NEU2. This finding was also supported in this report by the inhibition study (Fig. 5) and by the expression study of *Aire* and sialidases (Fig. 4).

Now, we discuss the physiological function of Neu-medullocytes. We showed that some Neu-medullocytes express AIRE; thus, they are likely to be acting as APCs for negative selection. This hypothesis is supported by the evidence of B cells expressing *Aire* in the mouse^14^ or human thymus^15^, as previously discussed. Through further examination, we found that some of the AIRE^+^ mTECs that are known to be APCs exhibited X-NANA sialidase activity. How do Neu-medullocytes and mTECs differ in regards to physiological functions? It has been suggested that murine thymic B cells may enlarge the self-antigen spectrum, because there is only a very limited overlap in TRA expression patterns between mTECs and the other AIRE-expressing cells^14^. Indeed, several genes were expressed differently in human thymic B cells compared with their peripheral counterpart^15^. Although we need more quantitative and qualitative analysis on Neu-medulloctes, we could not do it at this moment because of the difficulty of the flow cytometric analysis of X-NANA-stained cells. Now the next important question is how the sialidases work in APCs and in the negative selection step; we hypothesize the following.

Step 1. Removal of sialic acids from T cells.

Tight interactions between APCs and T cells must be the first step of negative selection. To allow tight interactions, the removal of sialic acids from the cell surface must be required. Sialic acids of single positive T cells (SPTs), known to be more sialylated than double positive T cells^34^, can be removed by NEU1 sialidase on Neu-medullocytes as suggested by our recent study^8^.

Step 2. Tight interaction between APCs and T cells.

Although the primary functional components for the interaction are MHC on APCs and TCR on T cells, CD5 and/or Mac-1 on Neu-medullocytes acting as APCs can enhance the interaction after the removal of sialic acids from T cells. NEU1 on Neu-medullocytes can remove sialic acids from CD5 on T cells because (1) NEU1 and CD5 exist on Neu-medullocytes, as shown in this study, (2) NEU1 on mouse thymocytes removes sialic acid from CD5^8^, and (3) the expression of CD5 is highest on SPT cells^8,35^. The desialylated CD5s on both cells can interact more easily, because the ligand of CD5 is CD5^36^. As the binding interaction between CD11b/CD18 and ICAM-1 is enhanced by endogenous sialidase activity^37^, Mac-1 (CD11b/CD18) on Neu-medullocytes and ICAM-1 on T cells^38^ will also be enhanced by the desialylation by NEU1. Although MHC classII α- and β-chains are also sialylated heterogeneously, as shown by two-dimensional electrophoresis^39,40^, NEU1 on Neu-medullocytes may remove the sialic acids from MHC in a cis-manner^4^, if necessary.

Step 3. Apoptosis or other functions of sialidase in negative selection.

As a result of the negative-selection test for MHC and TCR, if TCR reacts strongly with an MHC that has an ectopic tissue-specific antigen, then the autoreactive T cell undergoes apoptosis. NEU3 and NEU4 may help the process because the major functions of NEU3 and NEU4 have been suggested to be apoptosis^5^. *Neu3* has been shown to be expressed at high levels in the thymus, among the many tissues reported^4^, whereas expression of *Neu4* was low^4^, as we also observed in this study (Fig. 4). The contributions of NEU3 and NEU4 to the apoptosis of T cells should be clarified concretely in the future. It has also been suggested that AIRE-positive mature mTECs are postmitotic and undergo apoptosis^41^ and that the speedy apoptosis of AIRE^+^ mTECs may be a mechanism to promote the cross-presentation of the array of peripheral-tissue antigens they produce^41^. In this study, anti-AIRE antibodies detected many particles (AIRE^+^ particles) inside or possibly outside of the cells (Fig. 3M). These figures might indicate the apoptosis of AIRE^+^ cells.

Finally, the origin of CD5-positive Neu-medullocytes seems to be rather B1 cell (called CD5B cell) because Neu-medullocytes express Mac-1 (CD11b), unlike thymic B cells reported by Perera *et al*.^13^. B1 cells recognize bacterial polysaccharide^42,43^ but not protein peptide^44^, and have functions related to autoimmunity^45,46^ or have a role in autophagy for metabolic homeostasis and self-renewal^47^.

## Materials and Methods

### Mice

AKR (haplotype k, I-A^k^) and C57BL/6 (haplotype b, I-A^b^) mice (5 to 8 weeks old) were purchased from Japan SLC, Inc. (Hamamatsu, Shizuoka, Japan). All animal care and experimental procedures in this study were approved by Hokkaido University Animal Experiment Committee and were performed according to the guidelines for animal experimentation of the Hokkaido University. The number of mice used has been described in each figure.

### Chemicals

X-NANA and siastatin B were purchased from Peptide Institute, Inc. (Osaka, Japan), DANA was purchased from Sigma-Aldrich (St. Louis, MO). C9-BA-DANA was kindly provided by Drs. M. Kiso and H. Ishida of Gifu University. Collagenase and DNase I were purchased from Roche Diagnostics (Basel, Switzerland). UEA-1 (*Ulex europaeus* agglutinin, manufactured by J-Oil Mills, Inc.) was purchased from Cosmo Bio Co. (Tokyo, Japan) and labeled with FITC, according to the previously reported method^48^.

### Antibodies

The following antibodies were purchased: FITC-conjugated F(ab′)_2_ fragment of donkey anti-mouse IgG (H + L) (Jackson ImmunoResearch, West Grove, PA), anti-CD5 (Q-20, goat polyclonal IgG for mouse CD5 at the N-terminus, Santa Cruz Biotechnology, Inc., Santa Cruz, CA), anti-NEU1 middle region antibody (rabbit polyclonal antibody, Aviva Systems Biology (San Diego, CA)), Anti-AIRE (rabbit polyclonal IgG against AIRE (KLH-conjugated synthetic peptide derived from human AIRE, purified by protein A (cross-reactive species: human, mouse, rat), from Bioss (Boston, MA)). The secondary antibody was FITC-labeled goat anti-rabbit IgG (Jackson ImmunoResearch) or Rhodamine (TRITC)-conjugated donkey anti-rabbit IgG (H + L) (Jackson ImmunoResearch). Rhodamine Red^TM^-X (R.R.)-anti-mouse IgG (R.R.-conjugated AffiniPure F(ab′)_2_ fragment of donkey anti-mouse IgG(H + L)) and R.R.-anti-mouse IgM (R.R.-conjugated AffiniPure F(ab′)_2_ fragment of donkey anti-mouse IgM, μ chain specific) were purchased from Jackson ImmunoResearch. For FITC-anti-CD5, the anti-CD5 antibody described above was conjugated to FITC, as previous described^48^. An anti-mouse MHC class II (anti I-A^k^, 10-2-16 (monoclonal antibody) cell line was obtained from the American Type Culture Collection (Manassas, VA), and the culture supernatant was purified, as described previously^39^ and labeled with FITC, as described previously^48^.

### Thymus section

After the mice were sacrificed, the thymus was carefully removed and embedded in optimum cutting temperature (OCT) compound (Tissue-Tek, Miles Inc., Elkhart, IN), quickly frozen in liquid nitrogen, cryostat-sectioned to a 10- to 12-μm thickness (Leica, CM 3050 S), applied to silane-coated glass slides, and air dried^1^.

### Thymic stromal cell isolation by enzyme digestion and the preparation of six fractions for mRNA extraction

The mice thymuses were enzyme-digested according to the method reported previously^22^. To prepare the thymic stromal cells by enzyme digestion, more than two thymuses were used at once. Briefly, thymuses from freshly killed mice were dissected, trimmed of fat and connective tissue, and cut into small pieces in RPMI-1640 media. The mixture was gently stirred at 4 °C for 30 min and pipetted, and the medium was changed two or three times after the fragments settled to remove thymocytes. The thymocytes in the medium were pooled as total thymocytes. From the settled thymic fragments, an aliquot was taken as the residual fraction (**R**), and the remainder was then incubated in 5 ml of 0.125% (w/v) collagenase D with 0.1% (w/v) DNase I in RPMI-1640 media at 37 °C for 15 min, with gentle agitation using a Pasteur pipette every 5 min. The first enzyme mixtures containing isolated cells were removed (as **E1**) after the fragments had settled and was replaced with fresh enzyme mixture for further incubation. Finally, for the third enzyme treatment, the fragments were treated with 0.125% (w/v) trypsin in Ca^++^/Mg^++^-free HBSS for 15 min. The second and third mixtures were pooled as **E2** (primarily stromal cells, containing mTECs) and were used for X-NANA staining and subsequent immunochemical staining. Almost all of the fragments were digested by the second and third treatments. Total thymocytes, after an aliquot was removed (**T**), were further fractionated into peanut agglutinin (PNA)-aggregated cells (**A**) and PNA-unaggregated single cells (**S**), as described previously^31^. The six fractions (T, A, S, R, E1, and E2) were used for mRNA extraction and RT-PCR assays.

### X-NANA staining (*in situ* sialidase activity staining) and histochemical staining with fluorescein-labeled antibodies

#### Staining of thymus sections

X-NANA staining of the thymus section was performed according to the method described previously^1^. After that, the cryostat sections were immersed in the stop solution for X-NANA staining and were washed with PBS, and then the sections were treated with the fluorescein-labeled antibodies to detect molecules on B cells: FITC-anti-mouse-IgG_,_ FITC-anti-CD5, FITC-anti MHC class II (I-A^k^) or R.R.-conjugated AffiniPure F(ab′)_2_ fragment of donkey anti-mouse IgM.

To detect AIRE or NEU1 on Neu-medullocytes, a cryostat section stained with X-NANA was treated with anti-AIRE or anti-NEU1 antibodies, washed, and then treated with a FITC-secondary antibody simultaneously with R.R.-anti-IgG or R.R.-anti-IgM (Fig. 2I,II). Alternatively, Rhodamine (TRITC)-conjugated donkey anti-rabbit IgG (H + L) (secondary antibody) and FITC-conjugated F(ab′)2 fragment of donkey anti-mouse IgG (H + L) were used (Fig. 2III,IV and Fig. 5).

#### Staining of the thymic stromal cell-suspension

The **E2** fraction, described above, was used for Fig. 3, and the E1 + E2 fraction was used as the **E** fraction in Fig. 2(IV) and Supplementary Fig. S2(D–F and J–L). The cells were harvested by centrifugation in a small conical tube, suspended in a 1% paraformaldehyde solution for 1 min to prevent the outflow of the cytosolic enzyme NEU2 from the cells, washed with PBS, and treated in a 96-well flat plate with 25 to 40 µl of reaction buffer containing X-NANA^1^ overnight, at 37 °C in a moisture chamber. The cells were then washed with stop solution^1^, centrifuged and stained with the antibodies described in each figure legend.

### Effects of sialidase inhibitors on X-NANA staining

An aliquot of the **T**, **E1** and **E2** fractions, described above, were mixed and incubated with X-NANA reaction mixture^1^ in the presence of 30 µM each of the following: DANA^49^, C9-BA-DANA^50^, and siastatin B^51^.

### Confocal microscopy

Images were acquired on an Olympus Fluoview FV300 laser-scanning microscope system equipped with an argon laser (488 nm), a HeNe laser (543 nm), and a diode laser (405 nm) for excitation. To visualize X-NANA (sialidase activity staining), 430- to 460-nm emissions were collected after excitation at 405 nm. FITC (505- to 525-nm emissions) and rhodamine (565-nm emissions) signals were visualized in sequential mode by 488 nm and 543 nm excitation, respectively, and standard emission filters.

### Reverse transcription/polymerase chain reaction (RT-PCR)

RNA extraction and real time PCR were conducted as described previously^8^. Total RNA was extracted from the six cell fractions (T, S, A, R, E1, E2) described above. The PCR primers used for *Aire* were 5′-GAA GCT GTA CCC ACC TCT GG-3′ and 5′-GTG CTC ATT GAG GAG GGA CT- 3′. The other primers were the same as those used in a previous report^8^.

## Supplementary information

Supplementary Figures

## Data Availability

The datasets generated during and/or analyzed during the current study are available from the corresponding author on reasonable request.

## References

[1] Kijimoto-Ochiai, S., Doi, N., Matsukawa, H., Fujii, M. & Tomobe, K. Localization of sialidase-positive cells expressing Mac-1 and immunoglobulin in the mouse thymus. *Glycoconj. J*. **20**, 375–384 (2004). Erratum of this article. *Glycoconj. J*. **22**, 463 (2005). 10.1023/B:GLYC.0000033994.99464.ce 15238702

[2] Kijimoto-Ochiai S (2008). Low expression of Neu2 sialidase in the thymus of SM/J mice - existence of neuraminidase positive cells ‘Neu-medullocyte’ in the murine thymus. Glycoconj. J..

[3] Gossrau R, Eschenfelder V, Brossmer R (1977). 5-Brom-3-indolyl-α-ketoside of 5-*N*-acetyl-D-neuraminic acid a new substrate for the light and electron microscopic demonstration of mammalian neuraminidase. Histochem..

[4] Monti E (2010). Sialidases in vertebrates: a family of enzymes tailored for several cell functions. Adv. Carbohydr. Chem. Biochem..

[5] Miyagi T, Yamaguchi K (2012). Mammalian sialidases: Physiological and pathological roles in cellular functions. Glycobiology.

[6] Pshezhetsky AV, Hinek A (2011). Where catabolism meets signalling: neuraminidase 1 as a modulator of cell receptors. Glycoconj. J..

[7] Maurice P (2016). New insights into molecular organization of human neuraminidase-1: Transmembrane topology and dimerization ability. Sci. Rep..

[8] Kijimoto-Ochiai S (2018). Existence of NEU1 sialidase on mouse thymocytes whose natural substrate is CD5. Glycobiology.

[9] Issacson PG, Norton AJ, Addis BJ (1987). The human thymus contains a novel population of B lymphocytes. Lancet.

[10] Miyama-Inaba M (1988). Unusual phenotype of B cells in the thymus of normal mice. J. Exp. Med..

[11] Inaba. M (1990). Functional analyses of thymic CD5^+^ B cells. Responsiveness to major histocompatibility complex class II-restricted T blasts but not to lipopolysaccharide or anti-IgM plus interleukin 4. J. Exp. Med..

[12] Akashi K, Richie LI, Miyamoto T, Carr WH, Weissman IL (2000). B lymphopoiesis in the thymus. J. Immunol..

[13] Perera J, Meng L, Meng F, Huang H (2013). Autoreactive thymic B cells are efficient antigen-presenting cells of cognate self-antigens for T cell negative selection. Proc. Natl. Acad. Sci..

[14] Yamano T (2015). Thymic B cells are licensed to present self antigens for central T cell tolerance induction. Immunity.

[15] Gies V (2017). B cells differentiate in human thymus and express AIRE. J. Allergy Clin. Immunol..

[16] Nagamine K (1997). Positional cloning of the APECED gene. Nat. Genet..

[17] Aaltonen J (1997). An autoimmune disease, APECED, caused by mutations in a novel gene featuring two PHD-type zinc-finger domains. Nat. Genet..

[18] Mathis D, Benoist C (2009). Aire. Annu. Rev. Immunol..

[19] Anderson MS, Su MA (2011). Aire and T cell Development. Curr. Opin. Immunol..

[20] Kawano H (2015). Aire expression is inherent to most medullary thymic epithelial cells during their differentiation Program. J. Immunol..

[21] Yang SJ (2006). The quantitative assessment of MHC II on thymic epithelium: implications in cortical thymocyte development. Int. Immunol..

[22] Gray DH, Chidgey AP, Boyd RL (2002). Analysis of thymic stromal cell populations using flow cytometry. J. Immunol. Methods.

[23] Pereira ME, Kisailus EC, Gruezo F, Kabat EA (1978). Immunochemical studies on the combining site of the blood group H-Specific lectin 1 from *Ulex europeus* seeds. Archiv. Biochem. Biophysics.

[24] Abdul-Salam F, Mansour MH, Al-Shemary T (2005). The selective expression of distinct fucosylated glycoproteins on murine T and B lymphocyte subsets. Immunobiol..

[25] Yamaguchi Y, Kudoh J, Yoshida T, Shimizu N (2014). *In vitro* co-culture systems for studying molecular basis of cellular interaction between Aire-expressing medullary thymic epithelial cells and fresh thymocytes. Biol. Open.

[26] Figueroa F, Klein D, Tewarson S, Klein J (1982). Evidence for placing the Neu-1 locus within the mouse H-2 complex. J. Immunol..

[27] Liang F (2006). Monocyte differentiation up-regulates the expression of the lysosomal sialidase, Neu1, and triggers its targeting to the plasma membrane via major histocompatibility complex class II-positive compartments. J. Biol. Chem..

[28] Kijimoto-Ochiai S (2002). CD23 (the low-affinity IgE receptor) as a C-type lectin: a multidomain and multifunctional molecule. Cell Mol. Life Sci..

[29] Kotani K (2001). Cloning, chromosomal mapping, and characteristic 5′-UTR sequence of murine cytosolic sialidase. Biochem. Biophys. Res. Commun..

[30] Koda T, Kijimoto-Ochiai S, Uemura S, Inokuchi J (2009). Specific expression of Neu2 type B in mouse thymus and the existence of a membrane-bound form in COS cells. Biochem. Biophys. Res. Commun..

[31] Kijimoto-Ochiai S (2013). Possible association of Neu2 with plasma membrane fraction from mouse thymus exhibited sialidase activity with fetuin at pH 7.0 but not at pH 4.5. Microbiol. Immunol..

[32] Potier M, Lu Shun Yan D, Womack JE (1979). Neuraminidase deficiency in the mouse. FEBS Lett..

[33] Seyrantepe V (2004). Neu4, a novel human lysosomal lumen sialidase, confers normal phenotype to sialidosis and galactosialidosis cells. J. Biol. Chem..

[34] Reisner Y, Linker-Israeli M, Sharon N (1976). Separation of mouse thymocytes into two subpopulations by the use of peanut agglutinin. Cell Immunol..

[35] Azzam HS (1998). CD5 expression is developmentally regulated by T cell receptor (TCR) signals and TCR avidity. J. Exp. Med..

[36] Brown MH, Lacey E (2010). A ligand for CD5 is CD5. J. Immunol..

[37] Feng C (2011). Endogenous PMN sialidase activity exposes activation epitope on CD11b/CD18 which enhances its binding interaction with ICAM-1. J. Leukoc. Biol..

[38] Fine JS, Kruisbeek AM (1991). The role of LFA-1/ICAM-1 interactions during murine T lymphocyte development. J. Immunol..

[39] Katagiri UK, Kijimoto-Ochiai S, Hatae T, Okuyama H (1989). Type analysis of oligosaccharide chains on human and murine MHC class II alpha chains by the lectin-nitrocellulose sheet method. Comp. Biochem. Physiol. B.

[40] Kijimoto-Ochiai S, Hatae T, Katagiri YU, Okuyama H (1989). Microheterogeneity and oligosaccharide chains on the beta chains of HLA-DR, human major histocompatibility complex class II antigen, analyzed by the lectin-nitrocellulose sheet method. J. Biochem..

[41] Gray D, Abramson J, Benoist C, Mathis D (2007). Proliferative arrest and rapid turnover of thymic epithelial cells expressing Aire. J. Exp. Med..

[42] Berland R, Wortis HH (2006). Origins and functions of B-1 cells with notes on the role of CD5. Annu. Rev. Immunol..

[43] Duan B, Morel L (2006). Role of B-1a cells in autoimmunity. Autoimmun. Rev..

[44] Parham, P. The immune system, 4th ed. (Garland Science, 2014).

[45] Youinou P, Renaudineau Y (2007). The paradox of CD5-expressing B cells in systemic lupus erythematosus. Autoimmun. Rev..

[46] Deng J (2016). B1a cells play a pathogenic role in the development of autoimmune arthritis. Oncotarget.

[47] Clarke AJ, Riffelmacher T, Braas D, Cornall RJ, Simon AK (2018). B1a B cells require autophagy for metabolic homeostasis and self-renewal. J. Exp. Med..

[48] Harlow, E. & Lane, D. Isothiocyanate labeling. In: Antibodies - a laboratory manual. 354–355 (Cold Spring Harbor Laboratory, 1988).

[49] Meindl P, Bodo G, Palese P, Schulman J, Tuppy H (1974). Inhibition of neuraminidase activity by derivatives of 2-deoxy 2,3-dehydro-N-acetylneuraminic acid. Virology.

[50] Magesh S (2008). Design, synthesis, and biological evaluation of human sialidase inhibitors. Part 1: Selective inhibitors of lysosomal sialidase (NEU1). Bioorg. Med. Chem. Lett..

[51] Rahman M (2015). Nobel pH-dependent regulation of human cytosolic sialidase 2 (NEU2) activities by siastatin B and structural prediction of NEU2/siastatin B complex. Biochem, Biophys, Reports.

