# Supplementary material for: Neu-medullocytes, sialidase-positive B cells in the thymus, express autoimmune regulator (AIRE)
Source: Sci Rep. 2019 Jan 29;9:858. doi: 10.1038/s41598-018-37225-y (PMC6351566; doi:10.1038/s41598-018-37225-y)
Supplement: Supplementary file 1 — Supplementary Figures [file 41598_2018_37225_MOESM1_ESM.pdf]

Supplementary Figures

Neu-medullocytes, sialidase-positive B cells in the thymus, express autoimmune regulator (AIRE)

Shigeko Kijimoto-Ochiai[1,2,*], Keiko Kamimura[1] and Toshiaki Koda[1,*]

[1] Faculty of Advanced Life Science, Hokkaido University, N21 W11, Kitaku, Sapporo 001-0021, Japan

[2] Present address: Life Space COSMOS, Hirosaki, 036-8222, Japan

* 1st Corresponding author (T.K.): E-mail: t-koda@sci.hokudai.ac.jp

Tel.: +81-11-706-9062, Fax: +81-11-706-9210

* 2nd Corresponding author (S.K.-O.): E-mail: cosmosko060@me.com

Tel. and Fax: +81-172-88-6636.

Supplementary Figure S1

Supplementary Figure S2

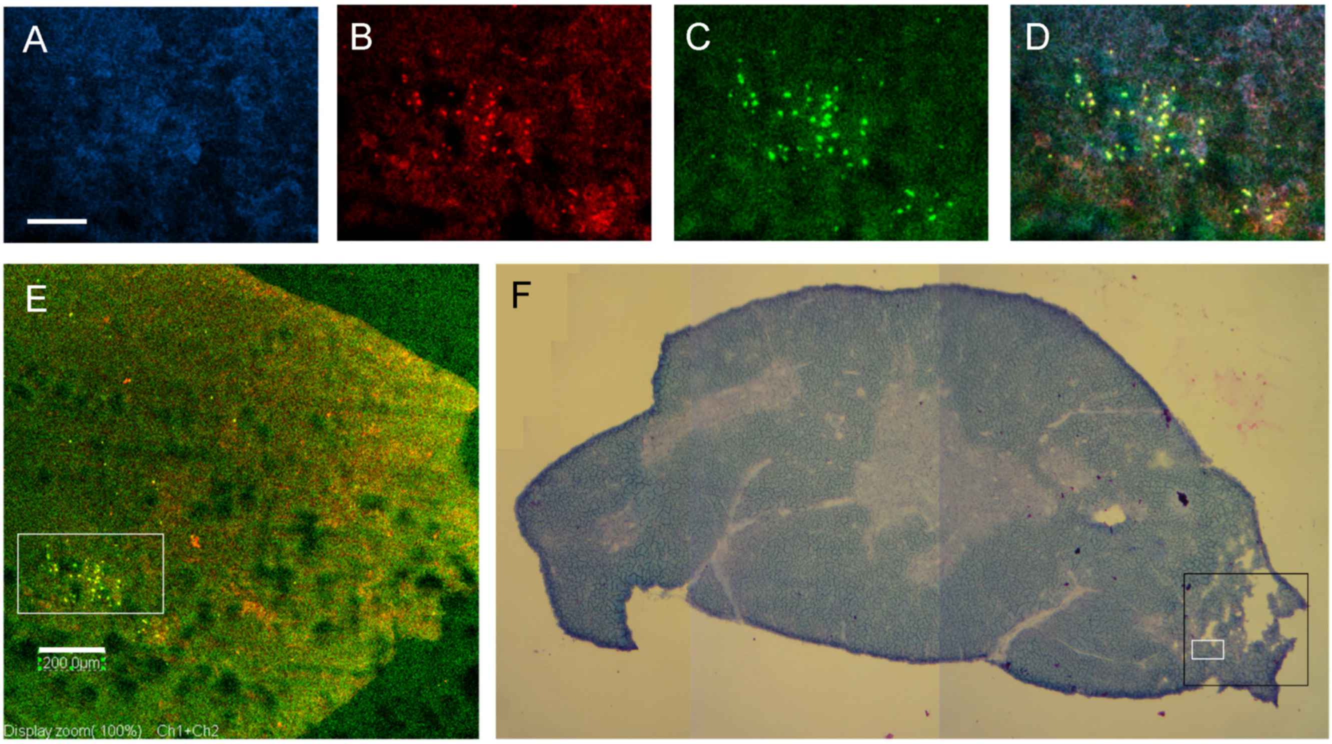

Supplementary Figure S1. IgG-positive cells in the thymus.

Thymus sections from an AKR mouse (4W, male) were stained with fluorescein-labeled antibodies and X-NANA and observed, X-NANA (**A**), R.R.-anti-mouse IgG (**B**), FITC-MHC class II (**C**) and merged (**D**). **E**, merged image of R.R.-anti-mouse IgG and FITC-MHC class II at lower magnification. **F**, Giemsa stained section, the black open square in F corresponds to the area of E. The white open square in E and F corresponds to the area of A-D. Scale bar in A is 100 µm for A-D. Scale bar in E is 200 µm.

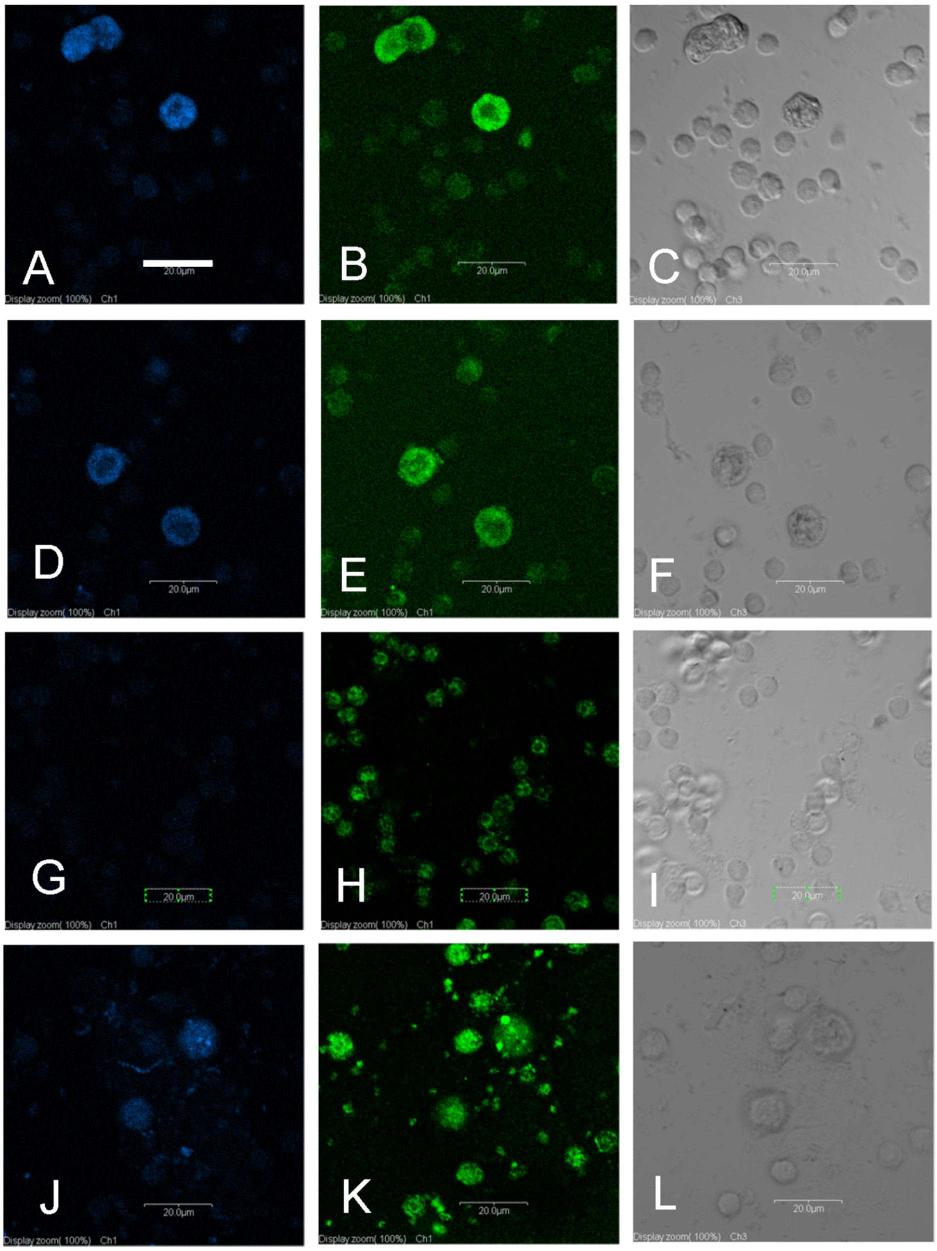

Supplementary Figure S2. The shapes of sialidase positive cells in enzyme treated fractions.

Cells were prepared from the thymuses of C57BL/6 (male, 6W) by enzyme treatment as described in the Methods section. The cells from the T (total thymocyte) or E (E1+E2) fraction were stained with X-NANA and FITC-anti-mouse IgG or with the FITC-anti-Aire system, and were observed as follows. **A-C**: T fraction, stained with X-NANA (**A**); anti-mouse IgG (**B**); or DIC (**C**). **D-F**: E fraction, stained with X-NANA (**D**); anti-mouse IgG (**E**); or DIC (**F**). **G-I**: T fraction, stained with X-NANA (**G**); FITC-anti-AIRE (**H**); or DIC (**I**). **J-L**: E fraction, stained with X-NANA (**J**); FITC-anti-AIRE (**K**); or DIC (**L**). Scale bars in A is 20 μm for all images.
